# Profile of follicle-stimulating hormone and polymorphism of follicle-stimulating hormone receptor in Madrasin cattle with ovarian hypofunction

**DOI:** 10.14202/vetworld.2020.879-883

**Published:** 2020-05-11

**Authors:** Budi Utomo, Emmanuel Djoko Putranto, Amaq Fadholly

**Affiliations:** 1 Department of Veterinary Reproduction, Faculty of Veterinary Medicine, Universitas Airlangga, Surabaya 60115, Indonesia; 2 Department of Veterinary Clinical, Faculty of Veterinary Medicine, Universitas Airlangga, Surabaya 60115, Indonesia

**Keywords:** follicle-stimulating hormone, follicle-stimulating hormone receptors, hypofunction, Madrasin

## Abstract

**Background and Aim:**

The follicle-stimulating hormone (FSH) gene is an essential regulator of fertility in livestock. This study aims to provide information on the genetic makeup of Madrasin cattle experiencing hypofunction by the FSH profile and FSH receptors (FSHR) polymorphism.

**Materials and Methods:**

Blood samples were collected from the Bangkalan regency in Indonesia. DNA was isolated and purified following the extraction protocol of polymerase chain reaction (PCR) and PCR-restriction fragment length polymorphism.

**Results:**

Our results showed that the FSH gene had a band length of 310 bp and produce two alleles (A and B) with restriction enzymes at 250 bp, 230 bp, and 145 bp. Furthermore, the FSHR gene had a band length of 303 bp and produced two homozygous genotypes: GG at bp 239 and CC at bp 188.

**Conclusion:**

Based on these differences, there was no change in allele frequency and genotype between Madura and Madrasin cattle due to crossbreeding with Limousin cattle. Thus, further detailed investigations of Madrasin cattle are required to elucidate the profile of the LH and LHR genes.

## Introduction

Madura cattle are beef cattle, considered as one of the richest Indonesian germplasm. Madura cattle are native to Madura Island and its surrounding islands. Morphologically, Madura cows possess almost the same characteristics as Bali cows, except for their smaller body and horn size. The skin color of Madura cows and bulls is browner than Bali cows, with a white part covering the area from the lower leg to the knee and some of the buttocks [1,2]. In addition, Madura cattle are more resistant to heat, as signaling pathways for protein kinase A, B, and C [3,4]. Changes in the molecular structure of the follicle-stimulating hormone receptor (FSHR) gene can cause desensitization of the FSH receptors in the cell membrane, decreasing the efficiency in the transmission of hormone signals. The FSHR gene also plays an important role in ovarian stimulation. Physiological evidence can be used to predict differences in FSHR function and the ovarian response to FSH. In addition, changes in the DNA sequence can affect the activation of the FSHR gene: Mutations in this region of the genome can affect ovarian folliculogenesis and consequently female reproductive performance [5,6].

Today, there are two known polymorphic sites in the gene structure of FSHR. The first polymorphism was found in the extracellular domain at codon 307 (position of the nucleotide sequence 919) which could be occupied by alanine (GCT) or threonine (ACT). The second polymorphism lies in the transmembrane domain at codon 680 (position of the nucleotide sequence 2039) which can be occupied by asparagine (AAT) or serine (AGT). In positions 919 and 2039 of the nucleotide sequences, there was a change in nucleotide bases from guanine to adenine. Both polymorphic sites are located at exon 10 and form two allele variations on FSH Thr307/Asn680 and Ala307/Ser680 [7,8]. In general, polymorphisms in exon l0 FSHR genes are single-nucleotide polymorphisms, such as Thr307Ala and Ser680Asn. These three polymorphic sites are missense mutations that do not have any effect on the phenotype. Asn residues contribute to FSHR glycosylation and are important in the process of post-translational receptors on the cell surface. On the other hand, the Ser residue converges during phosphorylation, which has the potential to cause receptor turn over [9].

Restriction fragment length polymorphisms (RFLPs) are all mutations which eliminate or create a new recognition sequence for restriction enzymes. Insertions, deletions, and substitutions occurring in the recognition area of a restriction enzyme cause its active site to no longer be recognized [10]. The RFLP method has been applied to detect quantitative traits loci in livestock. RFLP detection has been developed and used for linkage studies in cattle, chickens, and pigs. However, there has been no research of this kind on Madrasin cattle.

This study was conducted to determine the FSH profile and FSHR polymorphisms in Madrasin cattle and Madura cattle experiencing ovarian hypofunction.

## Materials and Methods

### Ethical approval

All procedures performed in this study were approved by the Ethical Committee of Faculty of Veterinary Medicine, Universitas Airlangga, Indonesia (reference number: 1.KE.119.07.2019).

### Blood sample collection

Blood samples were obtained from Madrasin cows that have no pregnancy more than 2 years. About 5 mL of blood and serum samples were collected from the jugular vein using a Venoject and Vacutainer tubes with EDTA (blood) and without EDTA (serum). Samples were then stored at 4°C until further analysis.

### DNA extraction

DNA was isolated and purified using the QIAamp Mini spin column DNA extraction kit. A total of 200 μL of blood samples were lysed by adding 200 μL of lysis buffer solution and 20 μL of proteinase K (10 mg/mL), the mixture was then incubated at 56°C for 60 min in a water bath shaker. After incubation, 200 μL of 96% ethanol were added to the solution and centrifuged at 8000*× g* for 1 min. DNA purification was undertaken using the spin column method after adding 500 μL of wash buffer I washing solution followed by centrifugation at 8000 g for 1 min. After the supernatant was removed, the DNA was then washed again with 500 μL wash buffer II and centrifuged at 14,000 g for 3 min. After the supernatant was removed, the DNA was then dissolved in 200 µL elution buffer and centrifuged at 8000× *g* for the extracted DNA to be collected and stored at −20°C until further analysis.

### Genotype frequency, polymerase chain reaction (PCR)-RFLP, FSH, and FSHR genes

The composition of the PCR reaction was conditioned using a reaction volume of 25 μL, consisting of 100 ng DNA, 0.25 mM primers, 150 μM dNTP, 0.5 Taq DNA polymerase, and 1× buffer. Samples were denatured at a temperature of 94°C for 2 min, followed by 35 subsequent cycles of denaturation at 94°C for 45 s each, with an annealing temperature of 65°C for 30 s (GH), followed by a final extension cycle of 72°C for 5 min. The final PCR product was then electrophoresed on 1.5% agarose gel with 1× TBE buffer, then visualized using a ultraviolet transilluminator. Alleles were determined by interpreting the band that takes the most form of migration to the anode pole as allele 1 and allele 2.

PCR products obtained from each target gene were then analyzed using RFLP through a restriction enzyme with a cutting site in the GH gene. Subsequently, 4 µL of DNA were added to 0.5 µL of PCR product, then incubated for 17 h at 37°C. The primers used to amplify the FSH and FSHR genes in Madrasin and Madura cattle were obtained from a gene bank.

### Statistical analysis

Samples from Madrasin cattle were compared based on the same size (marker) and the allele frequency was calculated and the FSH hormone profile was tabulated.

## Results

### PCR FSH gene

**Figure-1:**
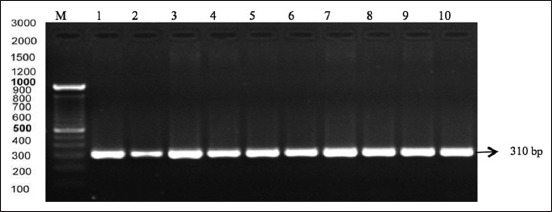
Polymerase chain reaction electrophoresis results with the primary Madrasin cattle follicle-stimulating hormone (FSH) gene suffering from hypofunction. Lane M: Marker, lanes 1-10 show the result of the electrophoresis of the Madura cattle FSH gene with a length of 310 bp.

The PCR results showed the presence of 10 FSH gene bands using the primary FSH gene. Positive results are shown in Figure-1.

### PCR-RFLP FSH gene

**Figure-2:**
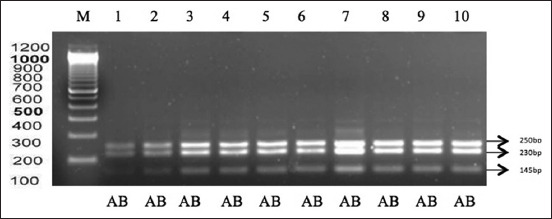
Electrophoresis results from polymerase chain reaction-restriction fragment length polymorphism using AluI retraction enzymes in the Madrasin cattle follicle-stimulating hormone gene. Lane M: Marker, lanes 1-14 (250 bp, 230 bp, and 145 bp).

The results of RFLP were 14 FSH gene samples divided into three bands, namely, 250 bp, 230 bp, and 145 bp. The results of the RFLP FSH for Madrasin cattle are shown in Figure-2.

### Genotype frequency and FSH gene allele

The results of the analysis of the Alul FSH gene segment show that each allele (A and B) has equal frequencies in Madrasin cattle (Table-1), while the genotype frequencies (AB and BB) were 1 and 0, respectively. This suggests that there was no change in allele frequency and genotype between Madura and Madrasin cattle after crossbreeding with Limousin cattle.

**Table-1:** Genotype frequencies and FSH gene alleles in Madrasin cattle (genotype and allele frequencies of Madrasin cattle).

Breed	n	Genotype frequency allele frequency				

		AA	AB	BB	A	B
Madrasin	10	0.00	1.00	0.00	0.50	0.50
Madura	10	0.00	0.00	1.00	1.00	0.00

AA, AB, and BB=Homozygous genotypes; A and B=Alleles. FSH=Follicle-stimulating hormone

### PCR FSHR gene

**Figure-3:**
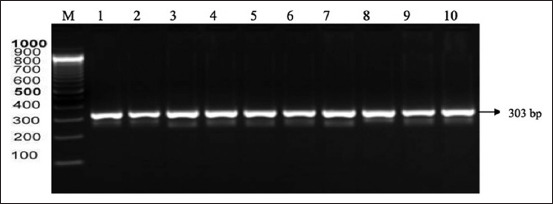
Polymerase chain reaction electrophoresis results with primary Madrasin cattle follicle-stimulating hormone receptor genes suffering from hypofunction. Lane M: Marker, lanes 1-10 show the results of the electrophoresis of Madura cattle rFSH gene with a length of 303 bp.

PCR amplification produced 14 bands of positive DNA samples using the primer FSHR gene. The positive visualization results from the electrophoresis are shown in Figure-3.

### PCR-RFLP FSHR gene

**Figure-4:**
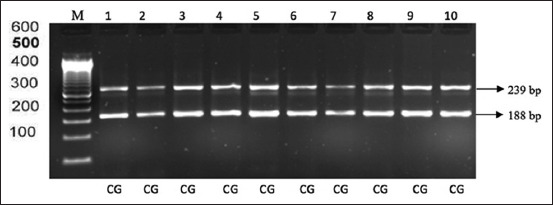
The electrophoresis results of polymerase chain reaction-restriction fragment length polymorphism with AluI restriction enzyme gene rFSH in Madrasin cattle. M: Marker, lanes 1-10 (239 bp and 188 bp).

After PCR products were digested with Alul restriction enzymes, two bands were obtained at 239 bp and 188 bp for Madrasin cattle. The electrophoresis results are shown in Figure-4.

### Genotype frequency and FSHR gene allele

The analysis results for the Alul FSHR gene segment show that frequencies for the C and G alleles are equal in Madrasin cattle (Table-2) while the genotype frequencies (CG and GG) were 1 and 0, respectively.

**Table-2:** Genotype frequency and rFSH gene allele in Madrasin cattle. Keterangan.

Breed	n	Genotype frequency allele frequency				

		CC	CG	GG	C	G
Madrasin	14	0.00	1.00	0.00	0.50	0.50
Madura	10	0.00	0.00	1.00	1.00	0.00

CC, CG, and GG=Genotype homozigot, C and G=Allele. FSH: Follicle-stimulating hormone

## Discussion

The FSH is secreted by the anterior lobe of the pituitary gland and plays an important role in female reproduction. FSH exerts its stimulatory effect by binding to FSH receptors (FSHRs) on granulosa cells in the ovaries and plays a key role in regulating fertility in livestock with high economic value. Therefore, the FSH gene could be a candidate gene in cattle [11,12]. Our results show that the amplification of the beta subunit FSH gene segment was successful at 310 bp (Genbank access number: J00008) [13].

The PCR-RFLP method used in this study is a widely used technique. PCR-RFLP allows researchers to identify homozygous and heterozygous individuals for each point mutation in the FSHR gene [14]. The FSH gene site of Madrasin cattle using the Alul retraction enzyme products along 250 bp, 230 bp, and 145 bp produced two alleles (A and B), while in Madura cattle, only allele B was found. Retraction enzymes can recognize the FSH gene at the cutting site, as DNA sequences at the cutting site do not mutate [15].

The results of the FSHR gene show that 303 bp of length and located at exon 10. After using PRC-RFLP, the FSHR gene segment amplified the genotypes for two Alul cutting sites (GG on one band: 243bp; CC at 239 bp and 188 bp). The FSHR gene fragment with the AluI enzyme cutting site indicates the absence of mutation [16]. The success rate of the FSHR gene amplification in this study was 100%. The temperature and time of annealing also determined the level of specificity of the amplification results. Furthermore, the quality (or purity) of the DNA used as a template was a determining factor for successful amplification [17].

The FSHR gene plays an important role in ovarian stimulation; thus, physiological evidence can be used to predict differences in FSHR function and ovarian response to FSH [18,19]. The hormones induce and maintain follicular development by binding to specific receptors on the surface of granulosa cells in the ovaries. This binding activates the gene coding for FSH to identify DNA polymorphisms in their relationship to productive and reproductive genotypes [20].

Diversity in the AluI FSHR gene segment was thought to be due to mutations or changes in the base causing changes in the amino acid serine to glycine. These changes cause the cutting site to become unrecognizable to the Alu1 enzyme [21,22]. Our study identified two types of cut fragments: The CG genotype at 239 bp and 188 bp and the CG genotype at 243 bp. Allele characterization of the FSHR gene in different cattle breeds provides us many advantages of heterozygosis and selecting individuals in loci of reproductive importance. The FSHR gene has an important role in ovarian stimulation and its physiology can be used to predict differences in the function of the FSHR and the ovarian response to FSH [23].

## Conclusion

Our study shows that the FSH gene in Madrasin cattle has a band length of 310 bp and produces two alleles (A and B) with restriction enzymes at 250 bp, 230 bp, and 145 bp. Furthermore, the FSHR gene had a band length of 303 bp and produces two homozygous genotypes (GG at bp 239 and CC at bp 188). Based on these findings, we assume that there was no change in allele frequency and genotype between Madura cattle and Madrasin cattle resulting from crossbreeding with Limousin cattle. Further investigations of Madrasin cattle are necessary to examine the profile of the LH gene and the LHR gene.

## Authors’ Contributions

BU contributed to conceptual design and performed experiments. EDP supervised the research. AF drafted and revised the manuscript. All authors read and approved the final manuscript.
